# Effect of savirin in the prevention of biofilm-related *Staphylococcus aureus* prosthetic joint infection

**DOI:** 10.3389/fphar.2022.989417

**Published:** 2022-09-15

**Authors:** Narayan Pant, Socorro Miranda-Hernandez, Catherine Rush, Jeffrey Warner, Damon P. Eisen

**Affiliations:** ^1^ College of Medicine and Dentistry, James Cook University, Townsville, QLD, Australia; ^2^ Australian Institute of Tropical Health and Medicine, Townsville, QLD, Australia

**Keywords:** *S. aureus*, savirin, mouse model, prosthetic joint infection, adjuvant therapy, arthroplasty

## Abstract

**Background:** Most of the arthroplasty surgery failure due to prosthetic joint infections (PJI) is caused by biofilm-associated *Staphylococcus aureus*. In a recent experimental study, savirin has been used to prevent and treat *S. aureus* skin infections in animal models. We explored the application of savirin in a PJI mouse model to determine its utility as an adjunct therapy to prevent PJI.

**Materials and methods:** The *in-vitro* antibacterial and antibiofilm activity of savirin, with or without antibiotics (cefazolin, rifampicin, and vancomycin), against *S. aureus* were investigated using broth microdilution and crystal violet staining method, respectively. The effect of savirin treatment on the expression of the key biofilm-related genes (*icaA, icaD*, *eno*, *fib*, *ebps*, and *agr*) in *S. aureus* was studied using quantitative reverse transcriptase polymerase chain reaction (qRTPCR). The *in-vivo* efficacy of savirin alone and with cefazolin to prevent *S. aureus* PJI was determined using a clinically relevant PJI mouse model. Mice were randomized into five groups (n = 8/group): 1) infected K-wire savirin treated group, 2) infected K-wire cefazolin treated group, 3) infected K-wire savirin plus cefazolin treated group, 4) infected K-wire PBS treated group, 5) sterile K-wire group. Savirin was administered subcutaneously immediately post-surgery and intravenous cefazolin was given on day seven.

**Results:** Savirin inhibited planktonic and biofilm *in-vitro* growth of *S. aureus,* showed enhanced inhibitory activity when combined with antibiotics, and down-regulated the expression of key *S. aureus* biofilm-related genes (*icaA, icaD*, *eno*, *fib*, *ebps*, and *agr*). Savirin significantly reduced bacterial counts on joint implants in comparison with the PBS treated control, while savirin plus cefazolin reduced bacterial counts on both implants and peri-prosthetic tissues.

**Conclusion:** Savirin adjuvant therapy may prevent biofilm formation and *S. aureus* PJI. This study gives baseline data for using savirin for the prevention as well as treatment of *S. aureus* PJI in future animal studies.

## Background

Indwelling medical devices, including prosthetic joints, create a favorable environment for biofilm-related bacterial infection (Ricciardi et al., 2018). Consequently, infection-related arthroplasty failure, mainly due to *S. aureus* infection, is common (Peel et al., 2012). Current treatments include major surgery either to replace or debride infected prostheses, both followed by long term antibiotic use (Zimmerli et al., 2004). However, these procedures have significant drawbacks - they are costly, potentially traumatic, and have failure rates ranging from 15 to 25% (Davis, 2016; Ma et al., 2018). Biofilm, a *S. aureus* growth mode that contributes to prosthetic joint infection (PJI) pathogenesis, is recalcitrant to antibiotic treatment (Morris et al., 2019). Therefore, antimicrobial therapy alone is not sufficient to treat most prosthetic joint infections (Del Pozo and Patel, 2009).

Savirin (*Staphylcoccus aureus* virulence inhibitor), is a low molecular weight, lipophilic, synthetic molecule suitable for drug development (Sully et al., 2014). This molecule prevents AgrA attachment to promoter regions in the *agr* quorum sensing system (Sully et al., 2014). It inhibits activation of the *agr* quorum sensing system, which is responsible for controlling many important *S. aureus* virulence factors, resulting in increased host-mediated bacterial killing (Sully et al., 2014). Savirin has been shown to prevent as well as treat biofilm-related *S. aureus* infections in rodent skin and subcutaneous infection models (Sully et al., 2014). Savirin might also be active against mature biofilm, as it was able to reduce infection even when administered 24–48 h post-infection establishment (Sully et al., 2014). Savirin was not toxic in doses (5 µg and 10 µg) used subcutaneously in two animal models (Sully et al., 2014). It appears that *S. aureus* is less likely to develop resistance to savirin than to conventional antibiotics as multiple *in-vivo* or *in-vitro* passages did not induce resistance in *S. aureus* to *agr* inhibition by savirin, while this did induce resistance to clindamycin (Sully et al., 2014).

There are limited data that characterize the antibiofilm efficacy of savirin and no previous study has investigated its use in the prevention of prosthesis-related infection caused by *S. aureus*. The objective of the current study was to investigate the effect of savirin*,* alone and in combination with antibiotics, on *S. aureus in-vitro* planktonic and biofilm growth and to determine the effect of savirin treatment on the expression of the key biofilm-related genes in *S. aureus*. Further, this study tested savirin’s effect as an adjuvant therapy for the prevention of *S. aureus* PJI in a mouse model.

## Materials and methods

A methicillin susceptible *S. aureus* clinical strain TUH_MSSA_01 isolated from a patient attending the Townsville University Hospital, Queensland, Australia was used in this study. *S. aureus* isolate was cultured in Luria-Bertani (LB) broth at 37°C for 48 h without shaking. Subculturing in 0.5% glucose containing LB (GLB) broth for 24 h induced ample biofilm production.

### *In-vitro* antibacterial and antibiofilm activity of savirin

Broth microdilution and crystal violet staining methods were performed in triplicates using microtiter plates. The 10^5^ cfu of *S. aureus* in 50 µL volume was added to eight two-fold serial dilutions of savirin (80 μg/ml to 0.62 μg/ml) in 50 µL volumes and incubated for 24 h at 37°C. This resulted in eight different savirin concentrations ranging from 40 μg/ml to 0.31 μg/ml. Antibacterial activity was determined by measuring the optical density (OD) of bacterial growth in microtiter plate wells at 600 nm. The minimum bactericidal concentration (MBC) of savirin was determined by plating the microtiter plate wells showing no bacterial growth for maximum 48 h. Microtiter plate biofilm assay procedures were adapted from a previous study (Singh et al., 2019). Planktonic bacterial culture in microtiter plate wells after 24 h of growth at 37°C in the presence of savirin was discarded and the biofilm formed was fixed with 2% sodium acetate for 10 min followed by overnight staining with 1% crystal violet. Absolute ethanol was used to reconstitute the crystal violet retained and absorbance was measured at 570 nm. *S. aureus* growth in the savirin diluent, DMSO (0.08%), was used as a positive control and the sterile DMSO (0.08%) was used as a negative control.

### Combined inhibitory effect of savirin and antibiotics (cefazolin, rifampicin, and vancomycin) on planktonic and biofilm growth

Savirin (26.67 μg/ml to 0.42 μg/ml) in combination with cefazolin (0.5 μg/ml to 0.007 μg/ml), or vancomycin (2.5 μg/ml to 0.03 μg/ml), or rifampicin (0.015 μg/ml to 0.0002 μg/ml) was used as described above, except with a total well volume of 150 µL (50 µL each of savirin, antibiotic, and bacterial broth culture suspension). The combined effect was tested by checkerboard assay by determining fractional inhibitory concentration (FIC) index value. The interaction of savirin and antibiotics was categorized as synergy (FIC < 0.5), antagonism (FIC > 4), and additive effect (FIC = 0.5–4) (Meletiadis et al., 2010; Alhashimi et al., 2019). Inhibitory effects of combined subinhibitory concentrations of savirin and antibiotics were also compared with use of each alone.

### The effect of savirin on expression of key biofilm-related genes in *S. aureus*

#### RNA extraction

RNA was extracted from log phase *S. aureus* culture treated with 10 μg/ml savirin (test sample) and 0.02% DMSO (control sample), using the Qiagen RNeasy mini kit. Ten µg/ml savirin was used, as this concentration reduced biofilm formation without inhibiting planktonic growth. The quality and quantity of RNA was determined using a Nanodrop 2000C spectrophotometer (Thermo Fisher Scientific, United States).

#### Gene expression quantification

Quantitative reverse transcriptase polymerase chain reaction (qRTPCR) was performed in triplicate for each gene by using Bio-Rad iTaq universal SYBR green one-step kit (Table 1). The reference gene used was *fema* because the expression of this gene was not affected when *S. aureus* was treated with savirin. The reaction mixture (10 µL) consisted of 5 µL of 2 × iTaq universal SYBR green reaction mix, 0.125 µL iScript reverse transcriptase, 0.8 ng RNA template in 1 µL volume, 1 nmol of primer mix in 1 µL volume, and 2.9 µL nuclease free water. The cycling conditions used on the BioRad CFX96 Touch Real-Time PCR Detection System were: reverse transcription (50°C, 10 min), polymerase activation and DNA denaturation (95°C, 1 min) followed by 40 cycles of denaturation (95°C, 10 s), and annealing/extension and plate read (60°C, 30 s). The effect of savirin treatment on the expression of key *S. aureus* biofilm-related genes, *icaA*, *icaD*, *eno*, *fib*, *ebps*, *agr* was determined by the comparative C_t_ (ΔΔC_t_) method using BioRad CFX Manager software (Livak and Schmittgen, 2001). The data obtained were expressed as fold changes (mean ± standard deviation) compared with control.

**TABLE 1:** Primers used for qRTPCR.

Primers	Oligonucleotide sequences (5' → 3′)	References
*icaA* (F)	CAA​TAC​TAT​TTC​GGG​TGT​CTT​CAC​TCT	Kot et al. (2018)
*icaA* (R)	CAA​GAA​ACT​GCA​ATA​TCT​TCG​GTA​ATC​AT	
*icaD* (F)	TCA​AGC​CCA​GAC​AGA​GGG​AAT​A	Kot et al. (2018)
*icaD* (R)	ACA​CGA​TAT​AGC​GAT​AAG​TGC​TGT​TT	
*eno* (F)	AAA​CTG​CCG​TAG​GTG​ACG​AA	Kot et al. (2018)
*eno* (R)	TGT​TTC​AAC​AGC​ATC​TTC​AGT​ACC​TT	
*ebps* (F)	ACA​TTC​AAA​TGA​CGC​TCA​AAA​CAA​AAG​T	Kot et al. (2018)
*ebps* (R)	CTT​ATC​TTG​AGA​CGC​TTT​ATC​CTC​AGT	
*fib* (F)	GAA​TAT​GGT​GCA​CGT​CCA​CAA​TT	Kot et al. (2018)
*fib* (R)	AAG​ATT​TTG​AGC​TTG​AAT​CAA​TTT​TTG​TTC​TTT​TT	
*agr* (F)	AAT​TTG​TTC​ACT​GTG​TCG​ATA​AT	Ferreira et al. (2013)
*agr* (R)	TGG​AAA​ATA​GTT​GAT​GAG​TTG​TT	
*fema* (F)	TGC​CTT​TAC​AGA​TAG​CAT​GCC​A	Francois et al. (2003)
*fema* R)	AGT​AAG​TAA​GCA​AGC​TGC​AAT​GAC​C	

#### Animal experiment

Ethics approval was obtained from the James Cook University Animal Ethics Committee (AEC2486). Six to 10 weeks old C57BL/6 female mice (Animal Resources Centre, Western Australia) were used. The mice were assigned to five experimental groups (8 mice/group): 1) infected K-wire savirin treated group, 2) infected K-wire cefazolin treated group, 3) infected K-wire savirin plus cefazolin treated group, 4) infected K-wire PBS treated group (positive control), and 5) sterile K-wire untreated group (negative control).

#### *S. aureus* prosthetic joint infection mouse model

Surgery was performed using previously described procedures (Bernthal et al., 2010). Mice were anesthetized with ketamine/xylazine (90 mg/kg/10 mg/kg, ip) prior to surgery. Buprenorphine (0.2 mg/kg, sc) was used as analgesic 30 min pre-surgery. Fur from the right thigh region was shaved followed by disinfection with povidone iodine. The skin was incised just above the knee and the patella was displaced to expose the tip of femoral bone. A hole was then made through the femoral intramedullary canal using a 26 G needle and a precut orthopedic-grade stainless steel Kirschner (K)-wire (diameter 0.6 mm) was inserted leaving a 1 mm protrusion into the joint space. A 2 µL *S. aureus* (TUH_MSSA_01) normal saline inoculum (500 cfu) was pipetted into the joint space. The kneecap was returned to its original position and the surgical site was closed with a 5–0 absorbable suture. A combination of subcutaneous (0.2 mg/kg) and oral (2.5 ml/160 ml drinking water) buprenorphine was administered for 72 h as post-surgical analgesia.

#### Savirin and antibiotic treatments

A single non-toxic subcutaneous dose of savirin (40 µg in 100 µL), as tested in the Vero cell line culture, was administered immediately after surgery to the infected K-wire savirin treated group. A single cefazolin (2.5 μg/g, iv) dose in 100 µL volume was administered on day 7 post-surgery to the infected K-wire cefazolin treated group. The infected K-wire savirin plus cefazolin treated group was administered a single dose of savirin (40 µg in 100 µL volume, sc) immediately after surgery followed by a single cefazolin dose (2.5 μg/g in 100 µL volume, iv) on day 7 post-surgery. Cefazolin was used in this manner not to sterilize the biofilm but to assess for any increased effect where it was used with savirin. Mice were weighed and animal well-being parameters, such as eating, drinking, mobility, interaction with other mice, and reaction to external stimuli were recorded daily. Mice were euthanized on day 14 post-surgery. The intramedullary K-wires were removed *in-toto* and peri-prosthetic tissues were collected aseptically for bacteriological culture.

#### Bacteriological analysis of K-wires and peri-prosthetic tissues

K-wires were collected in 5 ml of cold LB broth after washing three times with cold sterile PBS to remove planktonic cells. Sonication at 44 khz for 5 min using a waterbath sonicator was performed to thoroughly disrupt the attached biofilms. Similarly, tissues were collected in 800 µL of ice-cold PBS to minimize bacterial multiplication followed by homogenization using a Navy Lysis Kit (BioTools, Australia). Bacterial quantification of sonication fluids and tissue homogenates was performed by the drop dilution method whereby they were serially diluted and cultured on LB agar and mannitol salt agar (MSA) at 37°C for 48 h.

### Statistical analysis

One-way ANOVA was performed using GraphPad version 8.2.0 (GraphPad Software, San Diego, California, United States) followed by Tukey post-hoc test. *p*-value < 0.05 indicated statistical significance.

## Results

### Antibacterial and antibiofilm activity of savirin

**FIGURE 1:**
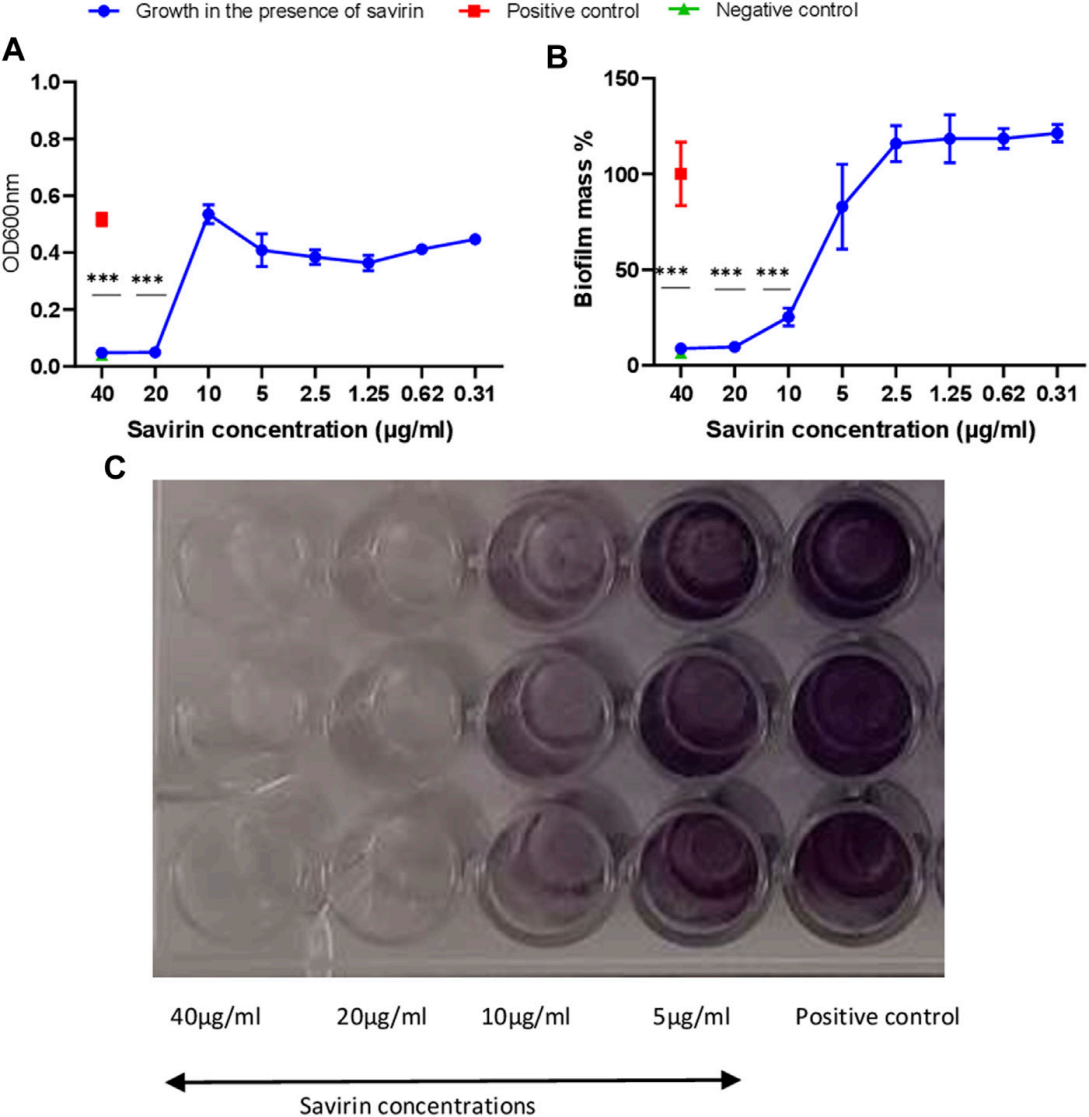
Planktonic **(A)** and biofilm **(B,C)** growth of *S. aureus* in the presence of different savirin concentrations. Triplicate wells were used for each treatment (N = 3) and experiments were repeated twice. Data are presented as mean ± standard deviation (SD) and the error bars indicate SD (*** = *p* < 0.001).

Savirin’s minimum bactericidal concentration (MBC) and minimum inhibitory concentration (MIC) were 40 μg/ml and 20 μg/ml respectively. Savirin at 40 μg/ml, 20 μg/ml and 10 μg/ml inhibited biofilm formation significantly (Figure 1).

### Combined antibacterial and antibiofilm activity of savirin and antibiotics (cefazolin, rifampicin, and vancomycin)

**FIGURE 2:**
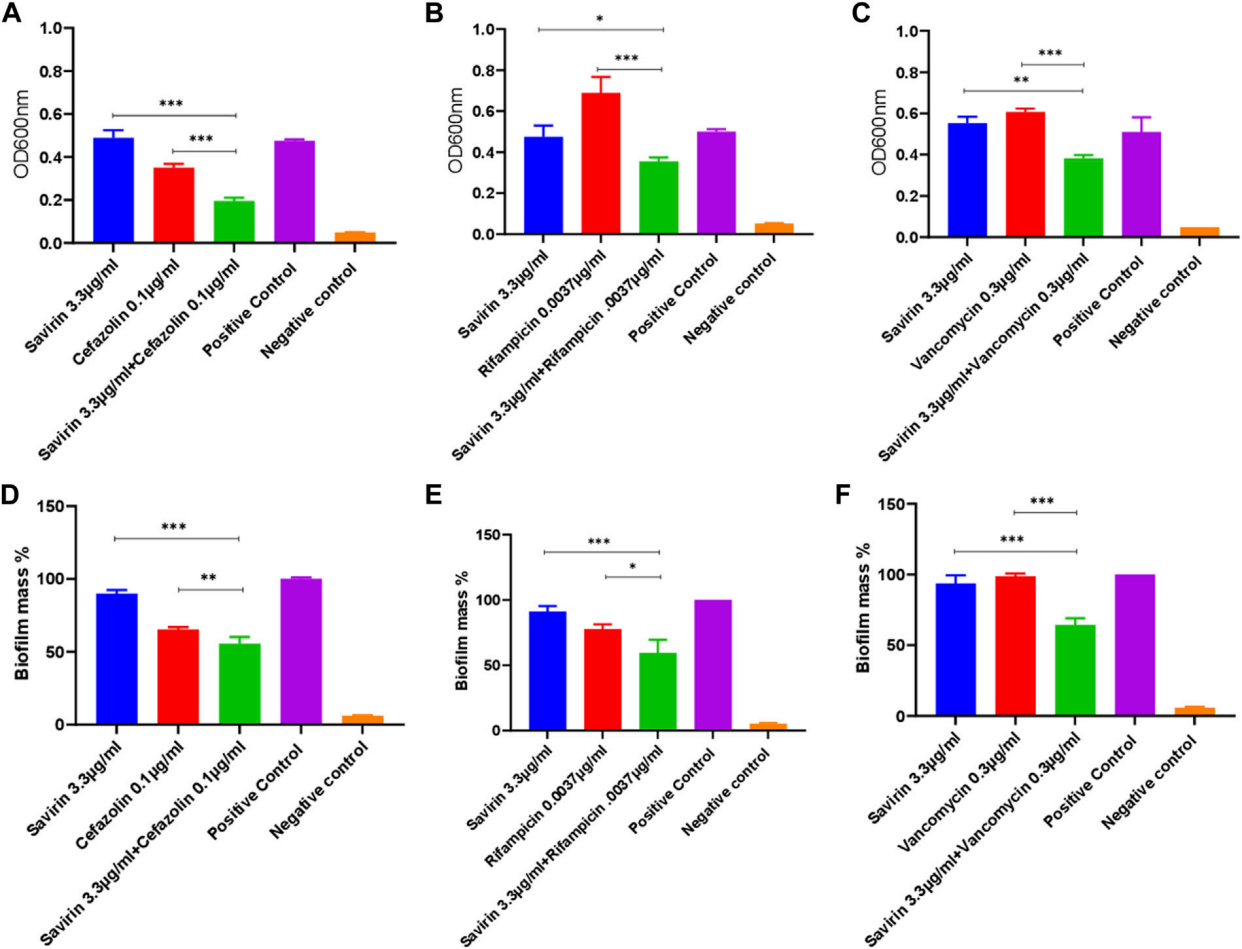
Inhibition of planktonic **(A,B,C)** and biofilm **(D,E,F)** growth by combined savirin and antibiotics (cefazolin, rifampicin, and vancomycin) sub-inhibitory concentrations compared with savirin and antibiotics alone (*** = *p* < 0.001, ** = *p* < 0.01, * = *p* < 0.05). Savirin concentration used in this experiment was sub-inhibitory. Savirin in combination with antibiotics showed enhanced antibacterial and antibiofilm activity against *S. aureus* compared with savirin alone. Experiments were performed in triplicates (N = 3) and data are presented as mean ± standard deviation (SD) with error bars indicating SD.

Multiple savirin and antibiotic concentration combinations were tested. The MICs of cefazolin, rifampicin, and vancomycin for the *S. aureus* strain used were 0.5 μg/ml, 0.015 μg/ml, and 2.5 μg/ml respectively. Combined sub-inhibitory concentrations of savirin and antibiotics showed significant enhanced antibacterial and antibiofilm activity compared with that of each alone (Figure 2). The fractional inhibitory concentration (FIC) index values for all three savirin and antibiotics combination ranged from 0.75 to 2 indicating an additive effect. The sub-inhibitory concentrations of savirin and antibiotics were chosen because they have no/minimal inhibitory activity when used alone such that enhanced combined inhibitory activity could be observed.

### Effect of savirin on the expression of *S. aureus* biofilm-related genes

**FIGURE 3:**
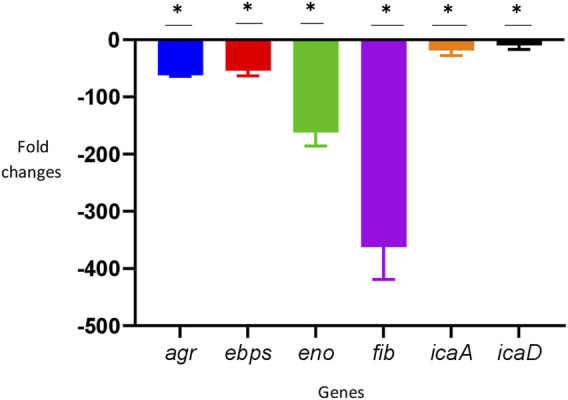
Downregulation of biofilm-related genes by savirin after 8 h of culture (* = downregulated > 2-fold). The reference gene used was *fema* and the experiment was performed in triplicate (N = 3). To determine the effect of savirin (10 ug/ml) treatment on the *S. aureus* biofilm-related genes, comparative C_t_ (ΔΔC_t_) method was used and the results were expressed as mean fold changes ± standard deviation (SD) in comparison with savirin diluent (0.02% Dimethyl Sulphoxide) treated control. The error bars indicate SD.

In the TUH_MSSA_01 strain, savirin downregulated all the biofilm-related genes tested significantly (> 2-fold) in comparison with the untreated positive control (Figure 3).

### Effect of savirin and/or cefazolin treatment on bacterial concentrations on K-wire implants and peri-prosthetic joint tissues

**FIGURE 4:**
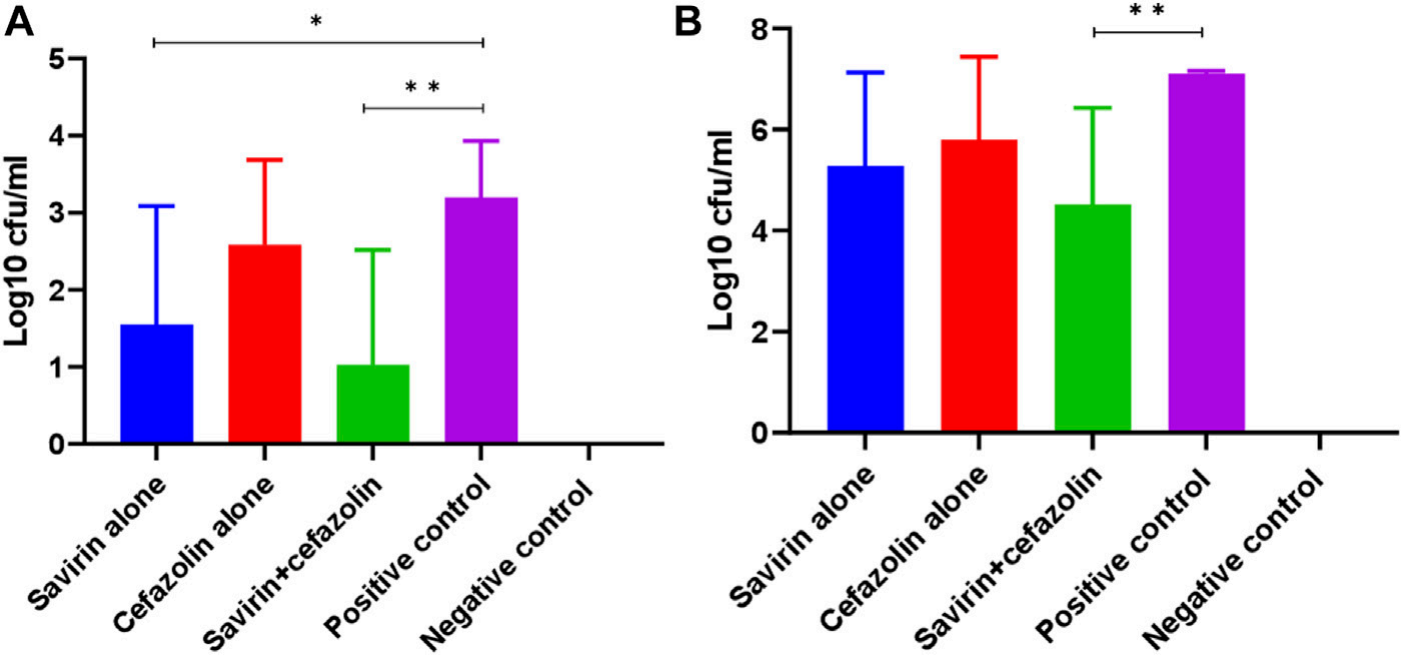
Bacterial counts on (N = 8) implant **(A)** and periprosthetic tissue **(B)** of different mice groups on day 14 post-surgery (** = *p* < 0.01, * = *p* < 0.05). Savirin alone reduced bacterial concentration on K-wire, while savirin plus cefazolin reduced bacterial concentration on both K-wire and periprosthetic tissue. The data are presented as mean log10 cfu/ml ± standard deviation (SD). The error bars indicate SD.

In this study, cefazolin showed better *in-vitro* activity when combined with savirin compared with other antibiotics tested. Additionally, this is the most common prophylactic antibiotic used in arthroplasty surgery. Therefore, cefazolin was chosen to use in the animal experiment over other antibiotics tested *in-vitro*. On animal well-being parameters testing, we did not report any adverse effects with the savirin concentration used in this study. Savirin significantly reduced bacterial counts on K-wires removed from the femur of mice with experimentally-induced prosthesis-associated septic arthritis in comparison with the PBS treated control (log10 cfu/ml, 3.2 versus 1.6) (*p* < 0.05). Similarly, savirin plus cefazolin reduced bacterial counts on both implants (log10 cfu/ml, 3.2 versus 1) and peri-prosthetic tissues (log10 cfu/ml, 7.1 versus 4.5) in comparison with the PBS treated control (*p* < 0.01) (Figure 4). The absence of an effect of cefazolin alone given on day 7 is keeping with persistence of *S. aureus* infection due to biofilm, indicating that this antibiotic failed to cure established biofilm.

## Discussion

Savirin showed the *in-vitro* antibacterial and antibiofilm activity, potentiated the *in-vitro* activity of selected antibiotics (cefazolin, rifampicin, and vancomycin) against *S. aureus*, and downregulated all the biofilm-related genes tested. In the PJI mouse model, this molecule prevented *S. aureus* infection.

This study reported the MBC and MIC of savirin for *S. aureus* to be 40 μg/ml and 20 μg/ml respectively. An earlier study also reported the direct *in-vitro* antibacterial activity of savirin (MIC = 36.8 μg/ml) (Mahdally et al., 2021). However, the mechanism of the action is unknown.

The inhibitory roles of savirin (5 μg/ml) against the *agr* quorum sensing system and a few other AgrA regulated genes, *hla*, *psm alpha*, *pvl* (*lukS*), have been reported previously (Sully et al., 2014). However, the same study showed no effect of savirin (5 μg/ml) treatment in the expression of other biofilm-related genes by microarray analysis (Sully et al., 2014). Consequently, inhibition of *agr*, a gene responsible for biofilm dispersal in *S. aureus,* by savirin (5 μg/ml) would have been expected to enhance biofilm formation (Vuong et al., 2000). However, while 5 μg/ml savirin had no effect, 10 μg/ml showed significant antibiofilm activity in this study. Downregulation of other biofilm-related genes might have negated the effect of *agr* disruption in *S. aureus* biofilm. Additionally, *agr* has strain-specific roles in staphylococcal biofilm formation and dispersal, and *agr* disruption might have increased, decreased, or no effect in biofilm formation in different strains (O'Neill et al., 2007; Yarwood and Schlievert, 2003). The discrepancy between the results of the previous and this study might be related to the higher savirin (10 μg/ml) concentration used in this study, and the different *S. aureus* strains and growth conditions used. The *ebps, eno,* and *fib* genes encode cell surface associated proteins and promote *S. aureus* adherence and colonization (Hartford et al., 2001; Downer et al., 2002; Tristan et al., 2003; Carneiro et al., 2004), while *icaA* and *icaD* induce bacterial slime production (Arciola et al., 2006). Therefore these genes are essential for the initial attachment of planktonic *S. aureus* cells to biofilm maturation and their downregulation may inhibit biofilm formation. In this study, savirin downregulated the *ebps, eno,* and *fib* genes more significantly compared with the *icaA* and *icaD* genes. Savirin perhaps prevents biofilm formation mainly by inhibiting *S. aureus* initial adherence and colonization then followed by prevention of extracellular matrix production. However, to determine the extent to which each genes' downregulation affected the biofilm formation, further studies using individual gene mutants strains are needed. In the prosthetic joint infection mouse model, savirin significantly reduced bacterial counts on K-wires and savirin plus cefazolin reduced bacterial counts on both implants and peri-prosthetic tissues in comparison with the PBS treated control. This indicates that savirin alone has *in-vivo* antibiofilm activity probably due to the prevention of *S. aureus* adherence to the K-wire prosthesis but no antibacterial activity. Savirin, instead, disarmed the bacteria by inhibiting biofilm, which were then cleared partially by cefazolin used on day 7. Savirin prevented the adherence of *S. aureus* to k-wire and monolayer formation by inhibiting the *ebps, eno,* and *fib* genes followed by further prevention of microcolony formation and biofilm maturation by inhibiting *ica* locus. There was no significant reduction in the bacterial counts in both the implants and tissues due to savirin plus cefazolin treatment compared with savirin alone treatment. This is inconsistent with the *in-vitro* results, where savirin plus cefazolin had significantly enhanced activity compared with savirin alone. It may be that the *in-vivo* diminished effect of savirin in our study relates to its rapid elimination before administering cefazolin to mice, leading to *S. aureus* biofilm formation to which cefazolin has limited activity. None of the treatments used in mice sterilized the implant or tissue infection even though the drugs showed complete *in-vitro* growth inhibition. This was probably due to the sub-inhibitory concentrations of savirin and cefazolin, to which *S. aureus* was being exposed *in-vivo*. However, more animal studies to determine the concentrations of savirin and cefazolin in the blood or knee joint tissues of mice, at different time points, are needed to reach definitive conclusions in this regard. This could also help to determine the optimal dose to sterilize infections in the mouse model.

There is only one other study that reported the influence of savirin on the prevention and treatment of *S. aureus* skin and subcutaneous infections in mouse models (Sully et al., 2014). In the previous study, savirin reduced infection even when administered 24–48 h post infection establishment implying its effectiveness against established *S. aureus* mature biofilms (Sully et al., 2014). Savirin doses of 5 µg and 10 µg were used to prevent and treat skin and subcutaneous tissue infections induced with *S. aureus* infectious doses 2×10^7^ to 4 × 10^7^ cfu (Sully et al., 2014). In this study, a higher savirin dose (40 µg) but a lower *S. aureus* infective dose (500 cfu) than the previous study was used and confirmed that savirin prevented infection. The infective dose used in the PJI model was chosen to establish a chronic septic arthritis, while the savirin dose was chosen because of theoretical concerns of reduced penetration into joints or bones.

Since the detailed animal toxicity profile, pharmacodynamics, and pharmacokinetics of savirin are not known, this study was unable to use higher savirin doses through different routes that might have sterilized the infection. Dose and toxicity finding studies including pharmacodynamics and pharmacokinetics studies are needed to allow for further animal model experiments. Testing of savirin in large animal prosthetic joint infection models that use materials and techniques used in a modern arthroplasty surgery is also recommended. These large animal models can better represent the real human infection pathogenesis compared with the simple mouse model used in this study.

*S. aureus* did not develop resistance against low concentration of savirin (5 μg/ml) in comparison with antibiotics (Sully et al., 2014). At 5 μg/ml, savirin does not directly inhibit bacteria but acts as a quorum sensing (QS) inhibitor and disarms bacteria exerting low selection pressure (Pan and Ren, 2009). However, the possibility of quorum sensing inhibitor resistance development among Gram-negative bacteria has been postulated (Defoirdt et al., 2010). Additionally, induction of dysfunctional *agr* has been reported, therefore the development of savirin (5 μg/ml) resistance through selection of *agr* dysfunctional mutants or stimulation of drug efflux requiring higher savirin concentration is possible (Somerville et al., 2002; Sully et al., 2014). Savirin’s binding site to AgrA includes a known mutation in *agrA* in human isolates mainly in strains colonizing the nose before the initiation of infection (Smyth et al., 2012). These *S. aureus agrA* mutant strains have been shown not to be efficiently transmitted between patients (Shopsin et al., 2010). With this information in mind it may be that *agrA* mutant *S. aureus* strains would not be a serious problem particularly in relation to PJI. However, there may be the possibility of resistance development of *S. aureus* against the direct inhibitory higher concentration of savirin and this needs to be investigated.

This study also explored the *in-vitro* antibacterial and antibiofilm activity of savirin against *S. epidermidis* (MIC = 40 μg/ml) and MRSA (MBC = 40 μg/ml, MIC = 20 μg/ml). Savirin was not active against *P. aeruginosa,* vancomycin resistant *Enterococcus*, and *Klebsiella pneumoniae* (detailed data not shown). These data indicate that savirin is, more broadly, an anti-staphylococcal agent with activity against both planktonic and biofilm growth forms. However, in the previous study no effect of savirin has been reported against *S. epidermidis* (Sully et al., 2014). This difference between results of the previous and this study might again be attributed to the higher concentration of savirin used in this study, and the different bacterial strains and growth conditions used in the two studies.

From our results, it can be concluded that savirin should be considered for the development of an adjuvant therapy for the prevention of *S. aureus* PJI. This study lays a foundation for studying this molecule for the prevention and treatment of *S. aureus* PJI.

## Data Availability

The original contributions presented in the study are included in the article/supplementary material, further inquiries can be directed to the corresponding author.
